# Digital cervicography and cold coagulation for cervical cancer screening in Nigeria

**DOI:** 10.1186/1750-9378-7-S1-P14

**Published:** 2012-04-19

**Authors:** Clement Adebamowo, Maryam Almujtaba, Zahra Modibbo, Olayinka Olaniyan, William Blattner

**Affiliations:** 1 Office of Strategic Information, Research and Training, Institute of Human Virology, Abuja, Nigeria; 2 Department of Epidemiology, Institute of Human Virology, University of Maryland School of Medicine, Baltimore, MD, USA; 3 National Hospital, Abuja, Nigeria

## Background

Cervical cancer (CC) is most common cancer among women in Africa and in women living with HIV [1,2]. Its prevalence has remained stable or increasing with introduction of HAART suggesting complex interactions with HIV [3,4]. Current screening programs can substantially reduce all-cause mortality of CC but implementation in LMIC is hobbled by poor infrastructure, cost and lack of personnel. Nurse provider led, minimal visit, screen and treat programs offer an opportunity to reduce CC morbidity and mortality in LMIC [5]. In this study we evaluate the implementation of cervical cancer screen and treat programs at 2 HIV treatment and prevention sites in Nigeria.

## Material and methods

CC screening programs using nurse providers, VIA, off the shelf camera for digital cervicography, treatment of eligible lesions by cold coagulation and referral as required was implemented at 2 PEPFAR supported sites in Abuja, Central Nigeria. QA was provided by Gynecologist and based on weekly review of digital cervicographs and client recall as required.

## Results

From July 2010 to July 2011, 2002 HIV+ women had been screened for CC at the 2 sites, but only data on 925 is reported in this abstract. Mean (SD) age was 35.2 (7.0) years; mean (sd) age at sexual debut was 19.0 (3.9) years; range, mean, sd of pregnancies was 0 – 16, 3.4, 2.5; range, mean, sd of pregnancies was 0 – 12, 1.6, 1.8; range, mean, sd of most recent cd4 count before screening was 11 – 1197, 466.7, 239.0; 6.8% were VIA positive; 0.2% had invasive CC and 0.2% were uncertain. Concordance between the clinical review and nursing diagnosis was 65% at the beginning of the program but reached 100% after 3 months.

## Conclusions

This study showed nurse provider led CC screening and treatment program is a viable public health intervention among PLWHIV in Nigeria.
